# Case Report: Therapeutic Use of Ibrutinib in a Patient With Schnitzler Syndrome

**DOI:** 10.3389/fimmu.2022.894464

**Published:** 2022-04-20

**Authors:** Yuehua Huang, Yanying Wang, Fan Yu, Xuehan Mao, Bianhong Wang, Jingxian Li, Lihong Li

**Affiliations:** Department of Hematology, Beijing Tsinghua Changgung Hospital, School of Clinical Medicine, Tsinghua University, Beijing, China

**Keywords:** Schnitzler syndrome, interleukin-1, Bruton tyrosine kinase inhibitor, ibrutinib, case report

## Abstract

Schnitzler syndrome is a rare adult-onset acquired autoinflammatory disorder typically characterized by chronic urticarial rash and immunoglobulin M (IgM) (rarely IgG) monoclonal gammopathy. Its clinical symptoms usually respond well to interleukin-1 blockade therapy, which, however, does not impact the underlying monoclonal gammopathy. Herein, we described a female patient who presented with urticarial rash, recurrent fevers, and fatigue for 7 years. Laboratory investigations revealed IgMκ monoclonal protein and *MYD88* L265P mutation, but no lymphoplasmacytic lymphoma on bone marrow examination. She fulfilled the diagnosis of Schnitzler syndrome and was treated with the Bruton tyrosine kinase inhibitor ibrutinib in combination with prednisone. Her symptoms improved dramatically, and the level of IgMκ monoclonal protein also declined. She tolerated the treatment well. This case highlights the potential therapeutic role of Bruton tyrosine kinase inhibitors in Schnitzler syndrome.

## Introduction

Schnitzler syndrome is a rare late-onset autoinflammatory disorder characterized by urticarial rash and immunoglobulin M (IgM) (rarely IgG) monoclonal gammopathy. Patients with this non-inherited disorder usually have disease onset in their 50s, with clinical features typically including recurrent urticarial rash, fever, bone pain, lymphadenopathy, and elevated inflammatory markers (1). Its clinical manifestations and therapeutic response to interleukin-1 (IL-1) blockade mimic the cryopyrin-associated periodic syndromes, which are caused by gain-of-function mutations in the *NLRP3* gene, a critical component of the NLRP3 inflammasome that is responsible for IL-1β production (1). Although IL-1 blockade therapy controls the symptoms well in most patients, it is not disease-modifying, so symptoms almost always relapse after discontinuation of the therapy. In addition, the underlying monoclonal gammopathy can progress while on treatment (2), which highlights the importance of exploring novel therapies. Bruton tyrosine kinase (BTK) inhibitors, such as ibrutinib, could target both the NLRP3 inflammasome and the underlying B-cell clone (3, 4). Herein, we report our successful experience of ibrutinib use in a patient with Schnitzler syndrome.

## Case Description

**Figure 1:**
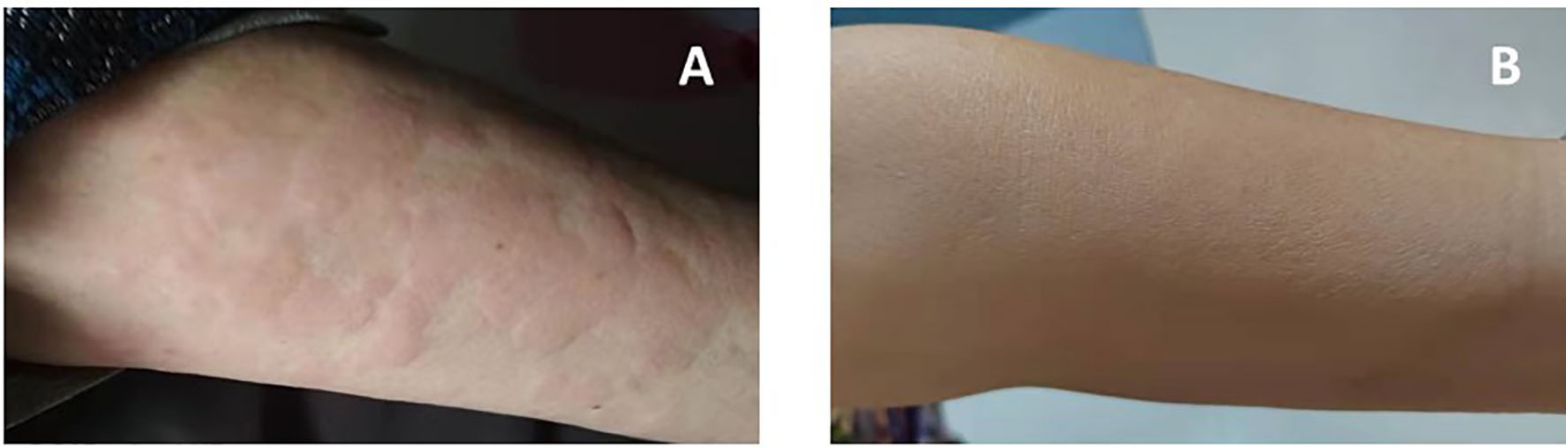
Resolution of urticarial rash after BTK inhibitor ibrutinib therapy [**(A)** pre-treatment; **(B)** post-treatment].

**Figure 2:**
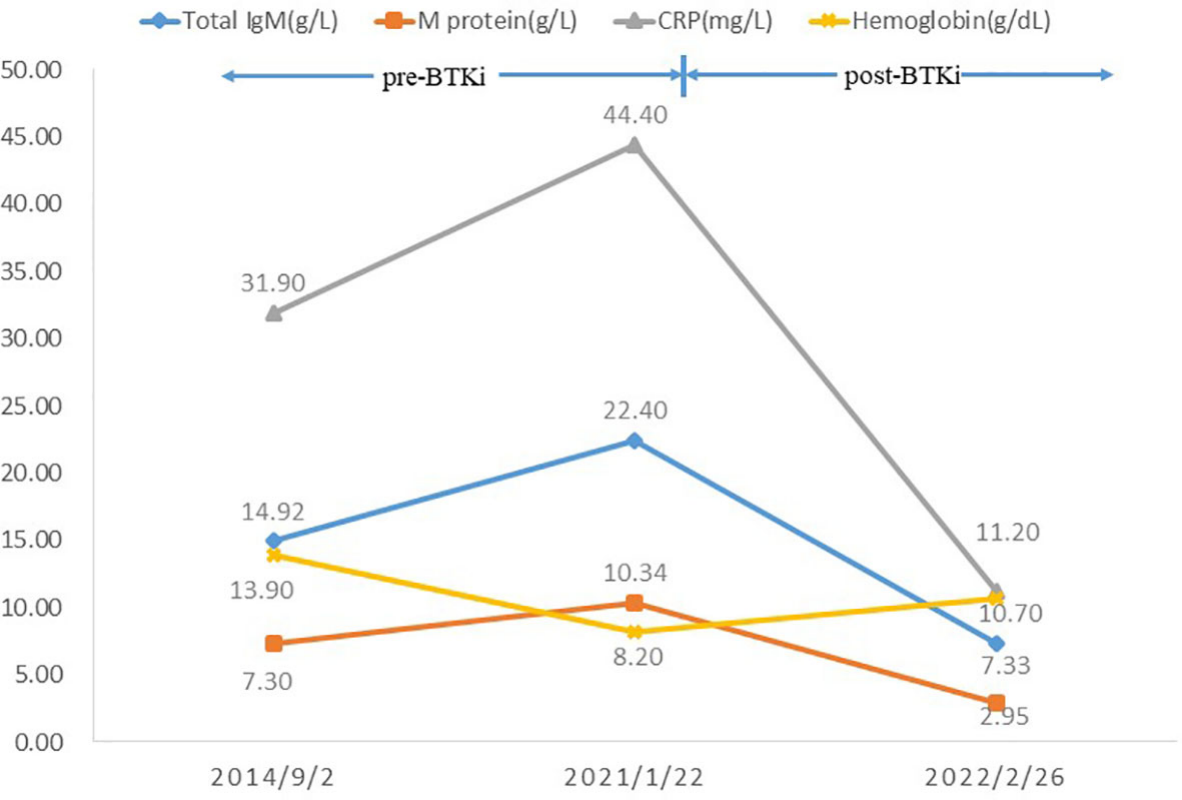
Trends of laboratory findings pre- and post-BTK inhibitor ibrutinib therapy.

A 71-year-old Chinese woman was admitted to our hospital in 2021 due to recurrent urticarial rash with fever and fatigue. These symptoms first started 7 years ago, and initial workups revealed mild leukocytosis, elevated levels of C-reactive protein (CRP) (31.9 mg/L; normal range, 0–10 mg/L), and IgM (14.92 g/L; normal range, 0.4–2.3 g/L). Serum protein electrophoresis showed a monoclonal band, but immunofixation was not performed. Otherwise, the patient did not have eosinophilia, and autoimmune panels, including anti-nuclear antibodies, anti-neutrophil cytoplasmic antibodies, anti-phospholipid antibodies, and cryoglobulin, were negative. The patient was started on prednisone 30 mg daily, and the dose was gradually tapered to 20 mg daily. However, her clinical response was unremarkable. On admission, she had intermittent fevers up to 39°C, and urticarial rashes without angioedema involving both trunk and extremities (**Figure 1A**). She was anemic (hemoglobin, 8.2 g/dl) with elevated CRP (44.4 mg/L) and serum IgM (22.4 g/L), but normal IgG and IgA levels. Serum electrophoresis quantified a monoclonal protein of 10.34 g/L, and immunofixation demonstrated an IgMκ subtype. Bone marrow examination did not show lymphoplasmacytic lymphoma morphologically, but flow cytometry revealed an abnormal population of B cells positive for CD5, CD19, CD20, CD22, surface IgM, and cytoplasmic kappa. Genetic testing found *MYD88* L265P mutation, but wild-type *CXCR4*. The imaging study did not show osteosclerosis. Her rashes were not typical for adult-onset Still’s disease, and her autoimmune studies were negative for other well-defined connective tissue diseases. Moreover, the bone marrow findings did not show evidence of multiple myeloma or Waldenstrom’s macroglobulinemia. Overall, this patient fulfilled the diagnosis of Schnitzler syndrome by meeting two obligate and two minor components of the Strasbourg criteria. Given difficult access to IL-1 blockade therapy and that the patient declined rituximab, she was started on ibrutinib 420 mg daily in combination with prednisone 25 mg daily for her debilitating symptoms. The urticarial rashes resolved completely (**Figure 1B**), and fatigue improved after 2 months of treatment. At the 12-month follow-up, her Schnitzler syndrome clinical activity score (a semiquantitative scale for rashes, pain, fever, and weight loss: 0, absent–rare; 1, moderate; 2, frequent–severe) decreased from 4 to 0 (5), and there was moderate improvement of her anemia (hemoglobin, 10.7 g/dl) and declines in the levels of monoclonal IgMκ (2.95 g/L) and serum IgM (7.33 g/L) (**Figure 2**). She tolerated the treatment well without infectious or cardiac complications, and her dose of prednisone was tapered to 15 mg daily.

## Discussion

Schnitzler syndrome is an acquired autoinflammatory syndrome. In view of its striking similarity to cryopyrin-associated periodic syndromes, many studies have focused on the pathogenic role of the NLRP3 inflammasome pathway. Indeed, Schnitzler patients with active disease showed clearly higher serum levels of pro-inflammatory cytokines (i.e., IL-6 and IL-18) and the extracellular apoptosis-associated speck-like protein with caspase recruitment domain aggregates (6). Moreover, peripheral blood mononuclear cells from Schnitzler patients were found to be hypersensitive to lipopolysaccharide-stimulated IL-1 production (7). Dermal mast cells, neutrophils, and keratinocytes might also be involved in pro-inflammatory cytokine production. However, recent studies using next-generation sequencing have not revealed classic germline or somatic pathogenic mutations in *NLRP3* and 32 autoinflammation-related genes in a large number of Schnitzler patients (6, 8). Although the mechanism remains obscure, these findings clearly indicate inflammasome activation in Schnitzler syndrome, and its pathogenic role is further corroborated by the therapeutic efficacy of IL-1 blockade. Anakinra and canakinumab control the symptoms and reduce inflammation successfully in most Schnitzler patients, but they are not disease-modifying, and treatment discontinuation almost always results in clinical relapse (2).

IgM monoclonal gammopathy is one of the hallmarks of Schnitzler syndrome according to the Strasbourg criteria. Besides IgM monoclonal gammopathy, genetic testing found an *MYD88* L265P mutation in this case. *MYD88* is at the crossroad of toll-like receptor (TLR), IL-1R, and B-cell receptor pathways and may participate in uncontrolled inflammation. The somatic *MYD88* L265P mutation was found in peripheral blood samples of 9 out of 30 Schnitzler patients, a similar frequency of IgM monoclonal gammopathy of undetermined significance (MGUS) (8). In addition, its 10-year progression risk into lymphoproliferative disorders, most notably Waldenstrom’s macroglobulinemia, is also similar to IgM MGUS (9). These observations support the incorporation of Schnitzler syndrome into the spectrum of monoclonal gammopathy of clinical significance in which organ damages are related to monoclonal protein or other paraneoplastic mechanisms rather than to the proliferation of the underlying B-cell clone (10). However, conventional therapies, such as rituximab, against the underlying B-cell clone did not always result in organ improvement, which makes Schnitzler syndrome different from other typical monoclonal gammopathies of clinical significance. Moreover, IL-1 blockade did not affect the levels of monoclonal proteins, and IgM MGUS can progress while patients are receiving IL-1 blockade (11). Overall, the link between IgM monoclonal gammopathy and inflammasome activation in Schnitzler syndrome is still missing.

As reviewed recently, TLRs, CD33, NLRP3, and BTK are all attractive therapeutic targets for diseases related to inflammasome activation (12). In addition, BTK has been validated as a therapeutic target in B-cell malignancy, most notably Waldenstrom’s macroglobulinemia and chronic lymphocytic leukemia (13). Of note is that two Schnitzler patients with prior exposure to IL-1 blockade therapy were treated successfully with the BTK inhibitor ibrutinib (**Table 1**) (14, 15), which may target both IgM monoclonal gammopathy and the NLRP3 inflammasome (4). These findings prompted our use of ibrutinib in the current case, and it did show dramatic clinical and biochemical responses. Compared to the two prior cases with concurrent hematologic malignancies, our patient only had IgM MGUS. These observations may highlight the pathogenic role of underlying B-cell clones in the development of Schnitzler syndrome and propose a possible universal therapeutic role of clone-directed therapy, such as BTK inhibitors. Whether patients respond differently according to the *MYD88* mutational status remains unknown, and further studies are required.

**Table 1:** Schnitzler syndrome patients treated with the Bruton tyrosine kinase (BTK) inhibitor ibrutinib.

Cases	Age (years)	Gender	Clinical manifestations	Monoclonal protein and underlying B-cell malignancy	Systemic treatments before BTK inhibitor	Current treatment	Outcomes
Jani et al. (14)	86	Male	Urticarial rashes	IgM (light-chain subtype not specified); lymphoplasmacytic lymphoma	Anakinra, rituximab	Ibrutinib 420 mg daily	Significant improvement of urticarial rashes (<10% of body surface involvement, much less than his presenting baseline)
Claves et al. (15)	60	Female	Fever, urticarial rashes, polyarthritis, weight loss, asthenia	IgMλ and IgGκ; splenic diffuse red pulp small B-cell lymphoma	Corticosteroids, anakinra, rituximab, canakinumab, chlorambucil, and splenectomy	Ibrutinib 420 mg daily	Complete disappearance of fever, urticarial rashes, arthritis, and monoclonal IgM. Her indolent lymphoma remained stable.
Current case	71	Female	Fever, urticarial rashes, and fatigue	IgMκ; IgM MGUS	Corticosteroids	Ibrutinib 420 mg daily with prednisone 25 mg daily	Resolution of fever and urticarial rashes, as well as improvement of fatigue.

## Data Availability Statement

The original contributions presented in the study are included in the article. Further inquiries can be directed to the corresponding author.

## Ethics Statement

Written informed consent was obtained from the participant for the publication of this case report.

## Author Contributions

YH and LL wrote the manuscript. YW, FY, XM, BW, and JL collected clinical data and generated results. All authors contributed to the article and approved the submitted version.

## Conflict of Interest

The authors declare that the research was conducted in the absence of any commercial or financial relationships that could be construed as a potential conflict of interest.

## Publisher’s Note

All claims expressed in this article are solely those of the authors and do not necessarily represent those of their affiliated organizations, or those of the publisher, the editors and the reviewers. Any product that may be evaluated in this article, or claim that may be made by its manufacturer, is not guaranteed or endorsed by the publisher.
